# Non-efficacy benefits and non-inferiority margins: a scoping review of contemporary high-impact non-inferiority trials in clinical cardiology

**DOI:** 10.1007/s10654-021-00820-x

**Published:** 2021-11-18

**Authors:** Maarten J. G. Leening, Karim D. Mahmoud

**Affiliations:** 1 Department of Cardiology, Erasmus MC – University Medical Center Rotterdam, PO Box 2040, 3000 CA Rotterdam, The Netherlands; 2 Department of Epidemiology, Erasmus MC – University Medical Center Rotterdam, PO Box 2040, 3000 CA Rotterdam, The Netherlands

**Keywords:** Non-inferiority, Randomized clinical trial, Statistics, Methods, Epidemiology, Cardiology

Randomized non-inferiority trials are designed to study whether an experimental treatment (or diagnostic test) is not unacceptably worse in terms of efficacy compared to the standard of care, whilst providing specific advantages over the standard of care justifying this comparison [1–3]. The magnitude by which the experimental treatment is acceptably less efficacious than the standard of care is commonly referred to as the *non-inferiority margin*. This pre-specified margin allows for constructing the null hypothesis stating that the experimental treatment is statistically significantly worse (i.e. inferior) than the standard of care. Rejecting this null hypothesis permits a conclusion of non-inferiority. The aforementioned specific non-efficacy advantages over the standard of care are referred to as *non-efficacy benefits*, and can for instance come in the form of lower probability of adverse events or complications, convenience of use, or lower costs.

The non-inferiority design lends itself particularly well to trials on de-intensifying or withholding treatment with known adverse effects. Elegant examples of such trials present both the implied non-efficacy benefit as a primary outcome (tested for superiority) and the potential loss in efficacy as a co-primary (or hierarchical secondary) outcome (tested for non-inferiority), hence aiming to demonstrate that de-intensified treatment comes with less adverse events balanced by acceptable losses in efficacy [4–7]. For example, consider a trial aimed to study whether withholding one of two antithrombotic medications prescribed after transcatheter aortic valve implantation, would result in less bleeding complications (primary endpoint tested for superiority; inferred non-efficacy benefit of de-intensifying antithrombotic treatment) without leading to an unacceptable higher number of thromboembolic events (secondary endpoint tested for non-inferiority) [5]. Although not so common, meta-analyses of trials have also employed non-inferiority designs to analyze aggregate data in order to answer non-inferiority questions with greater precision [8].

In this issue of the *Eur J Epidemiol,* Dr Acuna and colleagues make a case for ways to improve the reporting of non-inferiority trials by putting greater emphasis on non-efficacy benefits [9]. The work by Dr Acuna and colleagues provides a strong theoretical foundation with clear practical recommendations on how to implement non-efficacy benefits and non-inferiority margins into everyday reporting of non-inferiority trials. However, contemporary practices on this matter are less well documented. Hence, we aimed to evaluate the current scientific practice regarding the use and reporting of non-efficacy benefits and non-inferiority margins in clinical cardiology—a field of medicine with an ever increasing number of non-inferiority trials [3]. We focused on recent trials in high-impact journals as these generally serve as focal point for future studies to be modelled on.

## Literature search and data collection

We aimed to identify recent high-impact non-inferiority trials in the field of clinical cardiology. Hereto, we restricted to studies published (in print or online) from January 1, 2019 onwards in the following leading general medical and cardiology journals: the New England Journal of Medicine, The Lancet, the Journal of the American Medical Association, the British Medical Journal, the European Heart Journal, Circulation, and the Journal of the American College of Cardiology. Using PubMed (https://pubmed.ncbi.nlm.nih.gov/) we searched for “*non-inferiority*” or “*noninferiority*” in the aforementioned journals. References were screened for further evaluation based on title and abstract, aiming to identify randomized clinical trials employing a non-inferiority design in the field of clinical cardiology. Both authors extracted trial parameters from the full text manuscript (and online supplementary files if required so) by focusing on primary non-inferiority outcomes (or secondary outcomes only if primary outcomes were only tested for superiority). The literature search was last updated on October 9, 2021.

If not reported in the original publication, we calculated the non-inferiority margin in terms of relative risk based on outcome (i.e. event rate or measurement) in the standard of care group:

- Relative non-inferiority margin = [outcome + absolute non-inferiority margin] / outcome

We did this based on both the outcome in the standard of care group as anticipated during trial design, as well as based on the observed outcome in the standard of care group within the trial.

The proportional increase in relative non-inferiority margins due to overestimation of the anticipated outcome was calculated as follows:

- Proportional increase in relative non-inferiority margin = [observed relative non-inferiority margin—1] / [anticipated relative non-inferiority margin—1]

## Findings on non-efficacy benefits and non-inferiority margins

A total of 59 trials were included in our scoping review [4–8, 10–63].

### Non-efficacy benefits

In 69% (n = 41) of the articles non-efficacy benefits could be retraced (or clearly inferred). Among those 41 articles, a minority provided a rationale for the magnitude of the non-inferiority margin in relation to the non-efficacy benefit at hand.

Non-efficacy benefits were most commonly (n = 23, 39%) related to anticoagulation strategies or comparing interventions that require different downstream anticoagulation strategies. Associated non-efficacy benefits included having to use less anticoagulants (with accompanying lower bleeding risk) or convenience to use anticoagulants without the need for therapeutic monitoring [5–8, 14, 17, 21, 22, 24, 28, 32, 33, 36, 40, 41, 44, 48, 58, 62, 63]. Another common non-efficacy benefit was the use of less invasive techniques to diagnose or treat a variety of cardiac conditions (n = 9, 15%) [19, 21, 23, 25–27, 42, 45, 54]. Among remaining non-efficacy benefits, interventions aiming to reduce hospital stay were frequent (n = 7, 12%) [12, 15, 19, 21, 26, 53, 60].

### Non-inferiority margins

Most non-inferiority margins were expressed as event rates of clinical endpoints, such as death, cardiovascular events, or bleeding complications. Besides counting clinical endpoints, outcomes included restricted mean survival time to clinical events [41], chest tube drainage volume after coronary bypass surgery [60], number of blood transfusions after cardiac surgery [11], platelet activity parameters [39], coronary artery diameter stenosis [10], and specificity to diagnose significant coronary artery disease [4]. Non-inferiority margins were mostly defined as absolute increases of anticipated outcomes in the standard of care group (n = 39 endpoints, 63%), whereas within the remaining trials they were pre-specified as relative non-inferiority margins (n = 23 endpoints, 37%).

When anticipated outcomes in the standard of care group were reported (n = 55 endpoints), the magnitude thereof was overestimated compared to the actual observed outcomes in the standard of care group in 56% (n = 31 endpoints). Stratifying these results by type of non-inferiority margin (absolute versus relative) showed that outcome in the standard of care group was overestimated in 62% (n = 24 endpoints) amongst trials with absolute non-inferiority margins, and in 44% (n = 7 endpoints) amongst trials with relative non-inferiority margins (χ^2^ P = 0.23).

Overestimating the anticipated outcome in the standard of care arm whilst testing an absolute non-inferiority margin will result in a bias towards rejecting the null hypothesis (i.e. increasing the likelihood of claiming non-inferiority), since the corresponding relative non-inferiority margin will increase [2, 3]. For instance, one study comparing different aortic valve prostheses assumed that 30.8% of patients would have a complication within 30 days of implantation (i.e. anticipated outcome usual care) and an increase of up to 8.5% in complications would be acceptable (i.e. absolute non-inferiority margin; corresponding to a relative non-inferiority margin of 1.28 ([30.8 + 8.5] / 30.8)) [47]. However, in the trial only 9.6% of patients had a complication (i.e. observed outcome usual care) which resulted an actually tested relative non-inferiority margin of 1.89 ([9.6 + 8.5] / 9.6), thereby rejecting the non-inferiority null hypothesis whilst allowing for a threefold higher frequency of complications (relative 89% increase instead of the anticipated 28% increase). If a relative non-inferiority margin of 1.28 would have been tested, the likelihood of rejecting the null hypothesis of non-inferiority would have been markedly reduced by the lower than expected rate of complications observed (similar to a superiority trial with lower than expected event rates). The average increase in relative non-inferiority margin due to overestimation of the anticipated outcome (amongst trials with an absolute non-inferiority margin) was a factor 1.65, with outliers at 3.19 [13] and 3.21 [47] Such overestimation induces substantial bias towards claiming non-inferiority in these trials. This was explicitly acknowledged as a major limitation by several trials in our literature review (e.g. [13, 17, 25, 47, 62]).

## Specific considerations related to non-efficacy benefits and non-inferiority margins

### Uncertain or lack of non-efficacy benefit

We encountered a substantial number of non-inferiority trials without clear non-efficacy benefits or perceived non-efficacy benefits based purely on theoretical grounds. This was clearly seen among trials comparing coronary stents or implantable valves from different manufacturers [10, 20, 31, 37, 43, 47, 50, 56, 57, 59], which can be considered studies to evaluate *good substitutes* [3] and often allowed for remarkable high anticipated relative non-inferiority margins > 1.5. A major concern here is the risk of *biocreep* when subsequent generations of stents or valves are only tested for non-inferiority against previous generations and at each comparison a loss of efficacy is permitted [3].

Non-efficacy benefits should not be claimed on purely theoretical grounds to justify some loss in efficacy. For instance, recent history has thought us that perceived non-efficacy benefits may actually not be present at all, or perform worse than usual care in studies comparing a bioresorbable vascular scaffold to drug-eluting coronary stents [64]. These scaffolds were designed to require a shorter window of antithrombotic medication after implantation, whilst having fewer rare late thrombotic complications seen with conventional stents. A number of trials demonstrated non-inferiority of scaffolds on early thrombotic complications during the window where less antithrombotic medication had to be used. However, long-term follow-up of these trials revealed that late thrombotic complications actually occurred more frequently with scaffolds compared to stents, which could only be mitigated by prolonged intensified antithrombotic medication use. So the journey of these scaffold started out with the assumption that less antithrombotic medication was needed compared to with stents, justifying non-inferiority studies at the time. In the end, longer antithrombotic therapy than is common with stents was required. Consequently, the manufacturer discontinued sales of these bioresorbable vascular scaffolds. This example serves as a lesson that non-efficacy benefits should not be claimed on theoretical grounds, but must either be obvious (e.g. clear convenience of use or massive cost savings) or be scientifically proven (e.g. fewer adverse events).

From a conceptual standpoint, the usual care arm of a non-inferiority trial should encompass a treatment or diagnostic test with established merits [3]. However, we encountered 6 trials with a placebo arm as the usual care group [18, 29, 30, 34, 38, 55]. These trials were all mandated by regulatory bodies (e.g. FDA) to demonstrate cardiovascular safety of novel non-cardiovascular medication over placebo. This makes for a unique counterintuitive design aiming to study whether non-efficacy outcomes remain below a certain acceptable limits (i.e. the non-inferiority margin), with the previously proven efficacy outcomes acting as non-efficacy benefits. Regulatory safety trials with active treatment in the usual care arm, instead of placebo, are generally easier to interpret [46].

Other than presence of any non-efficacy benefit to justify a non-inferiority study, the non-efficacy benefit should also be proportional to the allowed loss in efficacy (i.e. magnitude of the non-inferiority margin). Although this balancing act is often a matter of clinical judgement, for some tradeoffs empirical data may be available to inform decision making. For instance, when studying de-intensification of antithrombotic therapy, patient-reported quality of life after bleeding or thromboembolic events and associated health care costs are widely available. In our literature review, some of the trials tested expedited discharge algorithms for patients with chest pain in the emergency department. One trial demonstrated that such an algorithm could shorten the average stay in the emergency department by 3.3 h, whilst allowing a relative 71% increase in missed myocardial infarctions (i.e. anticipated relative non-inferiority margin 1.71) [53]. The other trial demonstrated a shortening of stay by 1 h, whilst allowing for a near threefold increase in missed myocardial infarctions (i.e. anticipated relative non-inferiority margin 2.67) [12]. It is highly debatable whether such large non-inferiority margins are clinically acceptable when put into perspective to the limited non-efficacy benefits.

### Composite endpoints including the non-efficacy benefit

A number of studies in our literature review combined non-efficacy benefits with loss of efficacy outcomes in a composite endpoint tested for non-inferiority, often referred to as *net clinical benefit* endpoints [5, 7, 13, 40, 44]. This is conceptually problematic, since the point of a non-inferiority trial is to balance non-inferiority gains against loss in efficacy. For example, one of these trials aimed to optimize the balance between thrombotic risk and bleeding one month after coronary stent implantation by halving the dose of an antithrombotic drug, expecting to lower bleeding risk (non-efficacy benefit), whilst accepting a relative 31% increase in clinical events (i.e. anticipated relative non-inferiority margin 1.31) [44]. These clinical events included a range of thrombotic complications and death, but also bleeding complications.

Combining non-efficacy benefits and efficacy outcomes into a composite endpoint dilutes the efficacy comparison, and assuming that non-efficacy benefits are present, can even balance out a unacceptable losses in efficacy that cannot be directly unmasked. This will bias the results towards rejecting the null hypothesis of non-inferiority. In the aforementioned trial, the lower dose of antithrombotic drugs was not only found to be non-inferior, but even superior to full dose for the primary endpoint [44]. Thrombotic and bleeding events were also reported separately, revealing that the primary endpoint was completely driven by a halving the number of major bleeding with low dose antithrombotic drugs. Thrombotic events were rare in both treatment groups to an extent that non-inferiority could not have been established if the primary endpoint would have been restricted to thrombotic events only (upper bound of the 95% confidence interval 1.45). This basically reduces the trial results to demonstrating that reduced dose antithrombotics comes with fewer bleeding complications, but it remains uncertain what the effects are on loss of efficacy.

### Crossovers and discontinuation

A trial participant switching experimental arm after randomization is referred to as a *crossover*. In superiority trials with intention-to-treat analyses, crossovers and participants stopping the experiment altogether will lead to a dilution of contrast between the experimental arms and hence bias towards the null hypothesis of no effect. However, in non-inferiority trials, a loss of contrast between the experimental arms bias towards rejecting the null hypothesis and hence should generally be avoided. For example, in a theoretical trial comparing two drug regimens where all participants stop taking the study drugs shortly after enrollment, it will be impossible to demonstrate superiority of one drug over the other, whereas both drugs will likely be declared non-inferior to each other. In our literature review, we encountered studies with disproportional larger number of crossovers towards usual care (up to 20-fold [52]) and trials with up to 40% of the participants discontinuing the study treatment [63], often reflecting real life clinical care. The interpretation of such non-inferiority trials is challenging and complementary adjusted per-protocol analyses are advisable to explore whether these results are congruent with the standard intention-to-treat analyses [3, 65].

### Incomplete outcome ascertainment

Incomplete follow-up for outcomes lowers event rates, hence biasing the trial towards claiming non-inferiority when absolute non-inferiority margins are used. An example of a tell-tale sign of incomplete follow-up is the unexpected low ratio of non-fatal myocardial infarctions to cardiac deaths [57].

## The road forward

The non-inferiority trial is a useful but vulnerable construct and clinicians and researchers should take a few key points in mind when considering non-efficacy benefits and non-inferiority margins in a trial. First, it should only be employed when non-efficacy benefits can be discerned with certainty, and balanced against potential accepted losses in efficacy (i.e. the magnitude of the non-inferiority margin) as described by Dr Acuna and colleagues [9]. Next, if non-efficacy benefits can be measured, non-efficacy benefits should ideally be the primary outcome tested for superiority and losses in efficacy as a co-primary (or hierarchical secondary) outcome tested for non-inferiority. Finally, in order to reduce the inherent vulnerability and hence avoid bias towards or away from non-inferiority due to unanticipated event rates, use of relative risk-based non-inferiority margins and event-driven follow-up rather than a fixed duration of follow-up is preferred from a methodological perspective.

Non-inferiority trials represent an evolving and increasingly frequent employed trial design in clinical cardiology research. Our scoping literature review shows the diversity in reporting and analytical standards with respect to non-efficacy benefits and non-inferiority margins in contemporary high-impact cardiology trials. These findings highlight the importance of awareness and guidance towards appropriate selection and standardized reporting of non-efficacy benefits and non-inferiority margins, in order to ensure that future non-inferiority trials will provide valid new insights and thereby improve care for patients with heart disease.

## Declarations

### Data transparency

Literature search results and subsequently extracted data are available upon reasonable request from the corresponding author.
